# Patent foramen ovale closure in elderly patients: Addressing challenges in real-world study and clarifying methodology

**DOI:** 10.1093/esj/23969873251369443

**Published:** 2026-01-01

**Authors:** Chi-Sheng Wang, Po-Lin Chen

**Affiliations:** Division of Neurology, Neurological Institute, Taichung Veterans General Hospital, Taichung, Taiwan; Institute of Brain Science, School of Medicine, National Yang Ming Chiao Tung University, Taipei, Taiwan; Division of Neurology, Neurological Institute, Taichung Veterans General Hospital, Taichung, Taiwan; Institute of Brain Science, School of Medicine, National Yang Ming Chiao Tung University, Taipei, Taiwan; Department of Post-Baccalaureate Medicine, College of Medicine, National Chung Hsing University, Taichung, Taiwan

**Keywords:** Patent foramen ovale, patent foramen ovale closure, elderly%

**To the editor,**

We thank Dr. Daghlas for the thoughtful comments. As a real-world study, baseline differences do reflect inevitable bias from clinical practice. As a result, we included the RoPE score, which comprises multiple indicators, as a covariate to minimize the bias from clinical practice. Although high-risk features of patent foramen ovale (PFO) were not included in the multivariable models to avoid overfitting, their impact was assessed in prespecified subgroup analyses as shown in Supplemental Figure 1 of our original article.^1^ PFO closure consistently favored better outcomes regardless of shunt size or high-intensity transient signals (HITS) burden. Our findings support and extend those of randomized controlled trials (RCT) by focusing on an older population.

In terms of conceptualization, our study was designed to evaluate PFO closure (PFOC) in elderly patients (⩾60 years) with cryptogenic stroke (CS) and confirmed PFO. All patients were assessed and enrolled based on a standard stroke pathway. Rather than pre-classifying patients as having PFO-attributable or incidental PFO, we aimed to provide outcome data in a population for whom robust evidence is currently lacking – older adults with CS and PFO.

Regarding the concern of immortal time bias, as shown in Table 1 of our article, the median time from the index event to PFO closure was relatively short (12 days), and we confirmed that no recurrent strokes occurred during this interval in our cohort.

Regarding the difference in follow-up duration, the PFOC group had a longer mean follow-up period (3.6 ± 1.8 vs 2.4 ± 1.6 years, *p* < 0.001) compared with the non-PFOC group. Which was primarily due to our predefined endpoint – recurrent ischemic stroke, which occurred more frequently in the non-PFOC group (5 in the PFOC group vs 26 in the non-PFOC group). Consequently, more patients in the non-PFOC group experienced outcome events earlier. Regarding treatment effects, we agree that dual antiplatelet therapy (DAPT) following PFO closure may have contributed transiently; however, in our study, DAPT was typically prescribed for 6 months post-procedure, followed by long-term single antiplatelet therapy. The non-PFOC group received best antiplatelet therapy based on prevailing guidelines at the time of enrollment, including some patients on DAPT, as previously reported in our earlier study.^2^ Given the extended mean follow-up period of over 3 years in the PFOC group, the transient effect of DAPT is expected to be minimized regarding long-term outcomes.

For outcome definitions, both index and recurrent ischemic strokes were diagnosed by neurologists based on standardized comprehensive evaluations. We thank Dr. Daghlas for highlighting this point and are pleased to clarify it here.

In addressing the diagnosis of paroxysmal atrial fibrillation (AF), we acknowledge that prolonged cardiac monitoring is essential in modern CS diagnostics. As noted in our discussion, this was the primary limitation. While our findings provide supportive real-world evidence, we agree that future RCTs with rigorous AF exclusion protocols are warranted to further refine treatment strategies.

Sincerely,


Chi-Sheng Wang, MD and Po-Lin Chen, MD


## Data Availability

Not applicable.
